# Vascular Anatomy of Segment IV of the Liver in Live Liver Donors

**DOI:** 10.7759/cureus.46281

**Published:** 2023-09-30

**Authors:** Shruthy K M, Rathi Sudhakaran

**Affiliations:** 1 Anatomy, A.C.S. Medical College and Hospital, Dr. M.G.R. Educational and Research Institute, Chennai, IND; 2 Anatomy, Amrita School of Medicine, Amrita Institute of Medical Sciences, Amrita Vishwa Vidyapeetham, Kochi, IND

**Keywords:** living donor liver transplantation, multi-detector computed tomography, segment iv, hepatic vein, hepatic artery

## Abstract

Background: Studies on the anatomy of the liver have helped surgeries such as liver resection. Liver resection is of significance in liver transplantation. In liver resection, the anatomy of segment IV is very important as it is more prone to ischemia.

Aim: The primary objective is to study the anatomical variations of the hepatic artery and hepatic vein of segment IV from MDCT images of the hepatic vasculature in living liver donors. This study aims to document the anatomy of the hepatic artery supplying segment IV and its venous drainage in 300 living liver donors.

Materials and methods: In this retrospective study, 600 MDCT images of hepatic vasculature were observed, and the interpretations were recorded. The origin of the artery to segment IV was documented. The observations of the hepatic vein were tabulated as classified in Nakamura’s study.

Results: Segment IV artery originates from the left hepatic artery (LHA) in 72% of the cases and the right hepatic artery (RHA) in 23%. Hepatic venous drainage of segment IV comprises type I, type II, and type III in 14.33%, 53.67%, and 30% of cases, respectively. Type I anatomy of the hepatic vein is preferred in both right and left lobe liver transplantation as the drainage from segment IV is safe.

Conclusion: Vascularity to segment IV is key in living liver donors, as donor safety is of utmost importance in the case of living donor liver transplantation.

## Introduction

Liver transplantation, as explained by Dilip [1], was once an experimental procedure that has evolved into a lifesaving operation for increasing the life span in patients with chronic liver diseases where medical interventions have reached their limits. Despite the advances in the management of chronic liver diseases, liver transplant has been the only prospect for long-term survival for the patients. The first living-donor liver transplant (LDLT) dates back to 1963 in the USA, and the cases reliant on LDLT have been on a steady rise. Donor safety is the highest priority in LDLT. A wide range of complications have been reported in the literature in donors of LDLT. Hence, a thorough preoperative donor evaluation is highly recommended to provide safety to the donor in LDLT. A clear understanding of the segmental and vascular anatomy facilitates successful liver resection. Donor selection is purely dependent on the clinical and radiological evaluation done preoperatively. Preoperative imaging of the donor’s liver guides a surgeon to identify an appropriate donor, thereby contributing to donor safety. Sahani expresses that a comprehensive amount of information about the vascular anatomy of the liver is obtained from multidetector CT images [2]. The vascular anatomy of segment IV in liver donors is documented in many Western and East Asian studies. This study focuses on the segment IV arterial supply and venous drainage in Indian living liver donors.

## Materials and methods

The retrospective study was done on 300 liver donors who underwent LDLT. The data were collected from live donors between the ages of 28 and 40. The data were collected from the Department of Radiology, Amrita Institute of Medical Sciences, Kochi, which comprises 600 images taken from liver donors. These images were taken for preoperative evaluation in donors who underwent hepatectomy from 2006 to 2014 and were collected from their preoperative evaluation records available in the Amrita MedVision software of AIMS. Images were taken using a 64-multidetector CT scanner (Siemens Sensation Cardia-64, Siemens Medical Solutions, Erlangen, Germany). The pre-contrast series was taken using a 5-mm slice thickness, and post-contrast series was taken after injecting 80 ml of low osmolar nonionic contrast medium (Omnipaque 350 mg) at a flow rate of 5 ml/second. The post-contrast series were taken at 6s, +20s, and +30s CT images for arterial and venous phases. The source images have undergone three-phase dual enhancement and are processed to maximum intensity projections (MIP) and reconstructed to volume rendering (VR) for final assessment and documentation. The interpretations from the MIP images were done with guidance from a gastrointestinal surgeon and a radiologist. The source of the segment IV artery is identified and recorded. The venous drainage to segment IV was classified according to the existing standard classification done by Nakamura (Table 1) [3]. The findings that are recorded are done to help surgeons in liver resection in LDLT.

**Table 1:** Nakamura’s classification of the hepatic venous drainage of segment IV LHV: left hepatic vein; MHV: middle hepatic vein

NAKAMURA'S CLASSIFICATION OF HEPATIC VENOUS DRAINAGE OF SEGMENT IV	
Type	Description
I	Segment IV drains predominantly into LHV
II	Segment IV drains predominantly into LHV and MHV
III	Segment IV drains predominantly into MHV

## Results

Segment IV artery

In the current study, the artery to segment IV originated mainly from the left hepatic artery (LHA) in 217 cases (72.33%). The right hepatic artery (RHA) supplies segment IV in 69 cases (23%). In 10 cases (3.33%), segment IV received its blood supply from both the LHA and RHA. Segment IV artery arose from a common hepatic artery (CHA) in two cases (0.67%) and an accessory hepatic artery in two cases. The hepatic vein draining segment IV was of type I in 43 cases (14.33%), type II in 161 cases (53.67%), and type III in 90 cases (30%) (Table 1).

The hepatic vein draining segment IV was of type I in 43 cases (14.33%), type II in 161 cases (53.67%), and type III in 90 cases (30%) (Table 2).

**Table 2:** Artery to segment IV LHA: left hepatic artery; RHA: right hepatic artery; CHA: common hepatic artery; ACC: accessory

ARTERY TO SEGMENT IV		
Name of Artery	No of Cases (N)	%
LHA	217	72.33%
RHA	69	23.00%
LHA+RHA	10	3.33%
CHA	2	3.33%
ACC	2	0.67%

**Figure 1:**
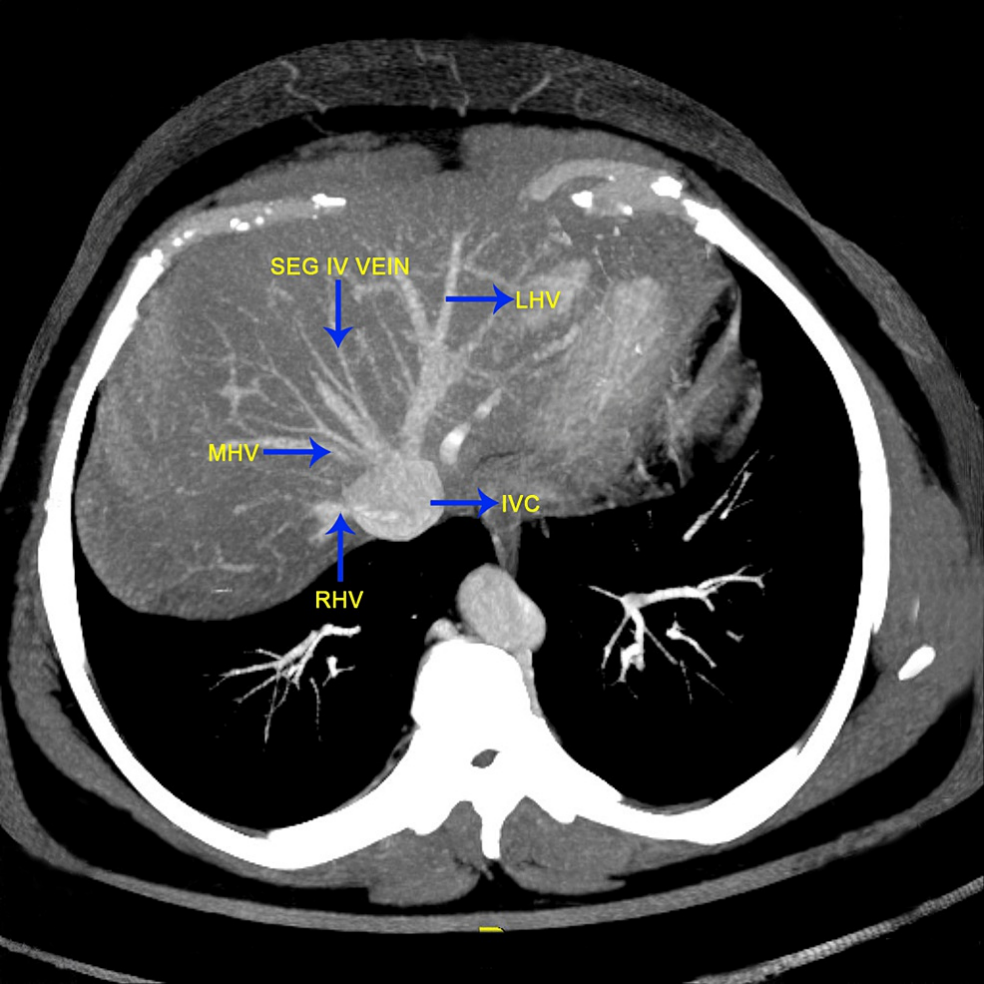
MDCT axial oblique view showing the segment IV drainage predominantly into the middle hepatic vein (type III) MDCT: Multidetector computed tomography; SEG IV: segment IV; MHV: middle hepatic vein; LHV: left hepatic vein (The figures are from the patients included in the current study.)

**Figure 2:**
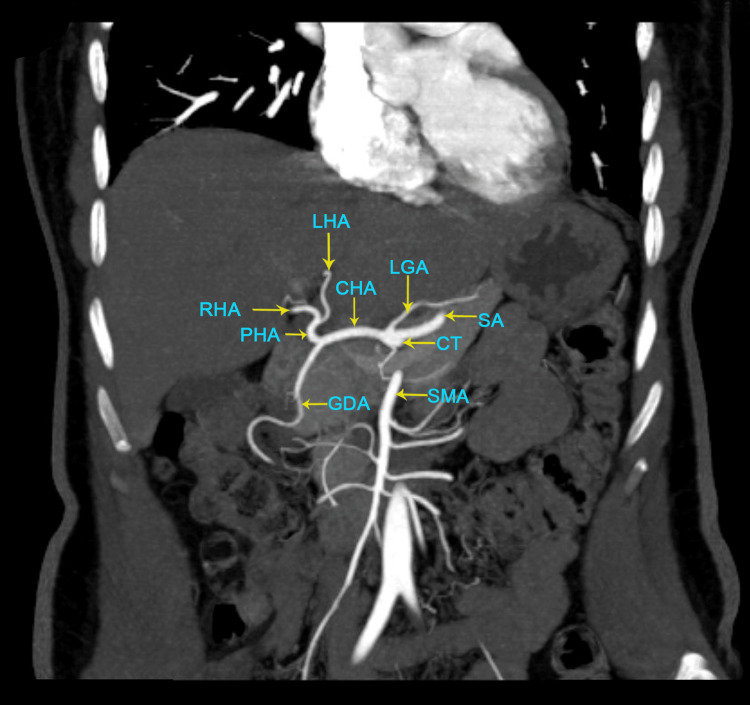
MDCT coronal MIP image of the normal hepatic arterial anatomy MDCT: multidetector computed tomography; MIP: maximum intensity projection; LHA: left hepatic artery; RHA: right hepatic artery; CHA: common hepatic artery; PHA: proper hepatic artery; LGA: left gastric artery; SA: splenic arteries; CT: celiac trunk; GDA: gastroduodenal artery; SMA: superior mesenteric artery (The figures are from the patients included in the current study.)

**Figure 3:**
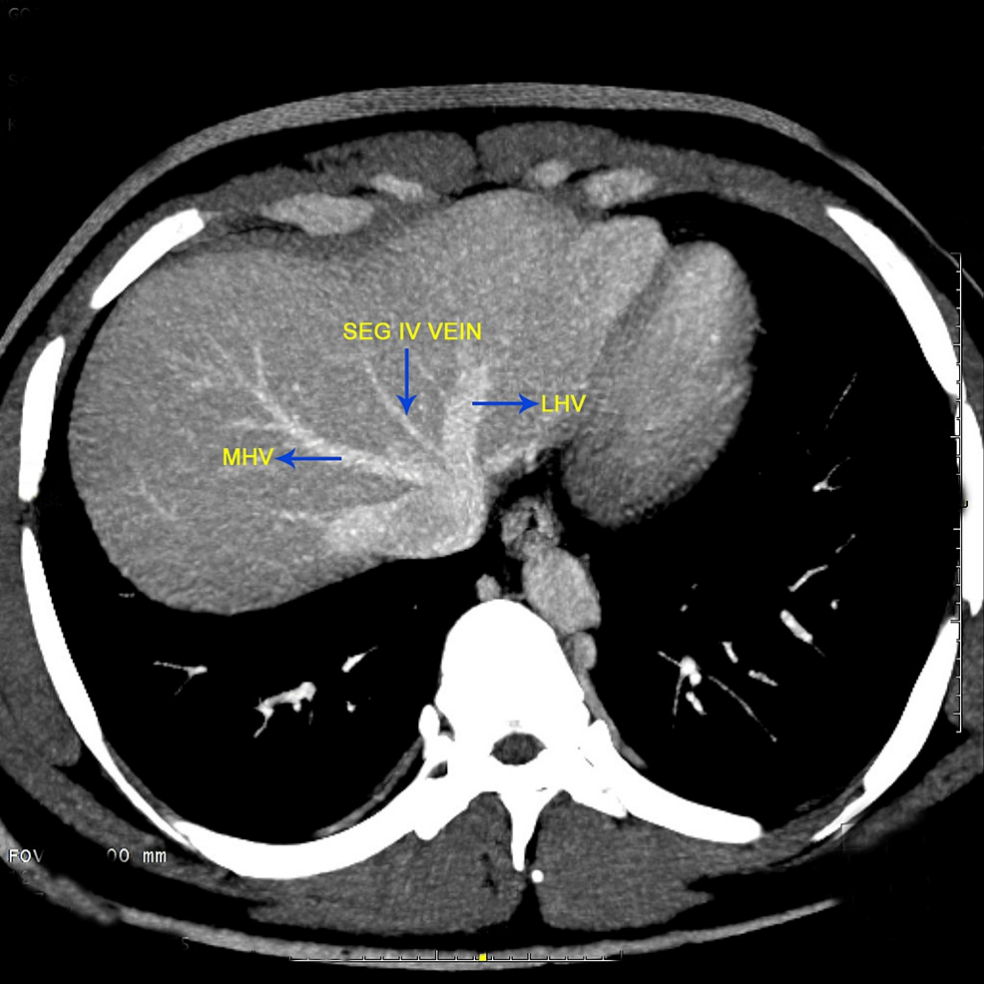
MDCT axial oblique view showing the segment IV drainage predominantly into the left hepatic vein (type I) MDCT: Multidetector computed tomography (The figures are from the patients included in the current study.)

The images given below show the MDCT axial oblique view showing the segment IV drainage predominantly into the middle hepatic vein type III (Figure 1), the MDCT coronal MIP image of the normal hepatic arterial anatomy (Figure 2), and the MDCT axial oblique view showing the segment IV drainage predominantly into the left hepatic vein type I (Figure 3).

Table 3 shows the number of cases in the current study that showed a variant arterial anatomy and hepatic venous drainage of segment IV in living liver donors.

**Table 3:** Hepatic venous drainage of segment IV

HEPATIC VENOUS DRAINAGE OF SEGMENT IV		
Type	No of Cases (N)	%
I	43	14.33
II	161	53.67
III	90	30.00%

## Discussion

The importance of liver resection is increasing from the perspective of treating several liver diseases. LDLT has given a new dimension to liver resection. Sahani states that the success rates in liver surgeries have been accomplished with evolving protocols in surgery, anesthesia, and critical care, in addition to better anatomical evaluation. Preoperative mapping of the liver and its vasculature helps the surgeon perform complex surgery with ease. Even the smallest detail of the hepatic vasculature will benefit the recipient and donors in LDLT. With the emergence of MDCT, it is preferred for assessing a potential liver donor [2]. The artery to segment IV is very important in transplant surgery as segment IV is spared in the surgery [3]. To assess the results in the current study, the hepatic artery is classified based on the source artery to segment IV, and segment IV venous drainage is classified according to a classification sourced from Nakamura’s [4].

In this study, LHA supplies segment IV in 72% (N=217) of cases. This finding is supported by studies done by Saylısoy et al., Taha Ali et al., Ahmed et al., and Elkholy et al. with a prevalence of 75% [5-8].

In 2005, Saylısoy et al. studied 52 potential liver donors in Turkey and reported that the artery to segment IV originated from the LHA in 39 patients (75%) and RHA in 13 patients (25%) [5]. Ali et al. (2012) in Egypt studied MDCT images of 43 potential donors and identified that, in 24 cases (75%), the artery to segment IV was from the LHA, and, in eight cases (25%), from the RHA [6]. Ahmed in 2012 did a retrospective study on 1,000 post-contrast CT scans of the abdomen. Segment IV was supplied from the LHA in 759 patients (75%), RHA in 203 (20%) cases, and CHA in 34 cases (3.4%) [7]. Elkholy et al. in 2013 evaluated 20 potential living donors with MDCT images and studied the dominant arterial branch to segment IV was a branch from the LHA in 15 cases (75%), whereas it branched from the RHA in five cases (25%) [8].

Kamel et al. (2001) did a study of MDCT images in 40 patients in Boston. In 25 (62.5%) cases, the RHA was the parent artery to segment IV [9]. Kishi et al. in 2004 studied MDCT images of 20 potential donors and reported that the dominant arterial branch to segment IV was a branch from the LHA in 15 cases (75%), and from the RHA in five cases (25%) [10]. Tsang et al. in 2008 dissected 62 livers and studied the segment IV artery. Segment IV artery took origin from RHA in 33 cases, LHA in 20 cases, PHA in two cases, and both the RHA and LHA in six cases [11]. Zhuang et al. in 2008 studied 102 potential liver donors who underwent CT angiography in China and found that the artery to segment IV was from the RHA in 23 donors (22.5%) and from the LHA in 77 (75.5%), and in two cases from a branch originated at the bifurcation of the PHA [12]. Ugurel et al. in 2010 did a retrospective study of the abdominal aorta and its branches in 100 patients from CT angiography in Turkey and documented that the artery to segment IV originated from the RHA in 35% of cases and 65% in the LHA [13].

In our study, the right hepatic artery sourced segment IV in 23% (N=69) of the cases. Segment IV received dual blood supply from both PHA and LHA in 10 of the cases. Zhuang et al. reported a dual supply to segment IV in six cases [12]. The CHA gave rise to the segment IV artery in 0.67% in the present study, and Ahmed et al. had reported a prevalence of 3.4% of the same [7]. The segment IV supply may be variable and, hence, their precise origin must be identified as these branches will cross the transection plane, especially when they arise from the RHA in cases of right lobe donation [1].

The hepatic venous anatomy is studied, giving importance to the drainage of segment IV. We obtained a significantly higher distribution of type II in 53.67% (N=161), while in Nakamura et al.’s study, it is 36% [4] and in Chan et al.'s study 36.7% [14]. Type III distribution is seen in 30% (N=90), whereas Nakamura et al. [4] and Chan et al. [14] obtained 5.8%. In our study, 2% were drained by an accessory vein that drained the segment IV directly into the IVC. The present study shows 14.33% (N=43) of the type I anatomy of venous drainage, whereas Nakamura et al. [4] and Chan et al. [14] reported a prevalence of 57%. Nakamura’s type I is the best-suited anatomy for liver transplantation.

The sample size gives an advantage to the study. Another strength of this study is that the anatomy of the hepatic artery and hepatic vein of segment IV identified in the MDCT images have been confirmed intraoperatively. This is also the first study conducted on living liver donors in an Indian setup. The only limitation of the study is that the study population includes only donors who underwent liver resection previously.

## Conclusions

In our study, we aimed to identify the common variants encountered in the hepatic vasculature targeted mainly on segment IV of the liver, specifically to living liver donors. In our study, most of the patient images showed that segment IV received its blood supply from the left hepatic artery, and the venous drainage of segment IV of the liver was by the middle hepatic vein and left hepatic vein. This study also aims to focus on how a detailed evaluation of the hepatic vascular anatomy in LDLT is essential to ensure successful postoperative results. The advent of triphasic CT protocol using a 64 multidetector CT scanner has shown a way to surgeons, especially transplant surgeons, where the anatomy of vasculature to the organ is of supreme importance for the postoperative viability of the organs received by the recipients. In the current study, a comprehensive and more accurate assessment of the detailed hepatic vascular anatomy in liver transplant potential donors prevents surgical complications arising from vascular variations.
